# Diversity in the Factors Associated with ADL-Related Disability among Older People in Six Middle-Income Countries: A Cross-Country Comparison

**DOI:** 10.3390/ijerph16081341

**Published:** 2019-04-14

**Authors:** Septi Kurnia Lestari, Nawi Ng, Paul Kowal, Ailiana Santosa

**Affiliations:** 1 Department of Epidemiology and Global Health, Umeå University, 90187 Umeå, Sweden; nawi.ng@umu.se; 2 Centre for Demographic and Ageing Research, Umeå University, 90187 Umeå, Sweden; ailiana.santosa@umu.se; 3 Research Institute for Health Sciences, Chiang Mai University, Chiang Mai 50200, Thailand; paul.r.kowal@rihes.org; 4 Vantage Health Solutions, Yangon 11072, Myanmar

**Keywords:** older adults, physical function, disability, ADL, WHO SAGE, LMICs

## Abstract

The low- and middle-income countries (LMICs) are experiencing rapid population ageing, yet knowledge about disability among older populations in these countries is scarce. This study aims to identify the prevalence and factors associated with disability among people aged 50 years and over in six LMICs. Cross-sectional data from the World Health Organization (WHO) Study on global AGEing and adult health Wave 1 (2007–2010) in China, Ghana, India, Mexico, the Russian Federation, and South Africa was used. Multivariable logistic regression analyses were undertaken to examine the association between sociodemographic factors, health behaviours, chronic conditions, and activities of daily living (ADL) disability. The prevalence of disability among older adults ranged from 16.2% in China to 55.7% in India. Older age, multimorbidity, and depression were the most common factors related to disability in all six countries. Gender was significant in China (OR = 1.14, 95% CI: 1.01–1.29), Ghana (OR = 1.22, 95% CI: 1.01–1.48) and India (OR = 1.65, 95% CI: 1.37–1.99). Having no access to social capital was significantly associated with ADL disability in China (OR = 2.57, 95% CI: 1.54–4.31) and South Africa (OR = 4.11, 95% CI: 1.79–9.43). Prevalence data is valuable in these six ageing countries, with important evidence on mitigating factors for each. Identifying determinants associated with ADL disability among older people in LMICs can inform how to best implement health prevention programmes considering different country-specific factors.

## 1. Introduction

Globally, the proportion of older people is increasing as a result of decreasing fertility rates and improvements in life expectancy [1]. About 80% of the world’s population aged 60 years and older will be living in low- and middle-income countries (LMICs) by 2050 [2]. Without adjustments to current policies and programme, demographic ageing has the potential to impact labour markets and workforces, taxation and pension systems, and health and social care systems, including family composition and living arrangements [3].

As of 2011, the World Health Organization (WHO) estimated that 15% of the global population is disabled [4]. More recent estimates are not available, but demographic ageing worldwide will contribute to disability levels. The number of older people with disability in LMICs is predicted to quadruple by 2050 [5]. Estimates from the 2002–2004 World Health Survey showed that disability prevalence among people aged 60 years and older was 43.4% in lower-income countries and 29.5% in higher-income countries [4]. Older people living with a disability are more likely to have low socioeconomic status, low education levels, poor social networks, be less engaged in the labour force, and have poor health [3].

Activities of Daily Living (ADLs) are one common measure used for estimating disability among older people [6]. The ADL questions assess people’s ability to perform activities such as eating, dressing, bathing, using a toilet, and getting in and out of bed [7]. Limitations in ADLs significantly predict the need for assistance, caregiving, nursing home placement, and health care utilisation [8,9]. Sociodemographic characteristics, health behaviours, chronic medical conditions, and social capital are significant predictors for ADL disability and mortality. Older age, female sex, low education, low socioeconomic status, being widowed or single, lack of access to social capital, sedentary physical activity, obesity, smoking, or having at least two chronic conditions (multimorbidity) are commonly associated with disability [10,11,12,13,14,15,16,17,18,19].

Much of the existing evidence regarding ADL related factors comes from studies conducted in high-income settings, while evidence from LMICs are scarce. The burden of disability in LMICs is predicted to increase as a consequence of the ongoing demographic transition towards an ageing population. Therefore, knowledge about the factors associated with disability is important for developing future health promotion and prevention programs in LMICs [20]. A few studies in LMICs have explored the association between sociodemographic factors, chronic diseases and disability [21,22,23]. However, comparable nationally representative estimates of disability and country-specific associated sociodemographic and health-related factors in LMICs is lacking [24]. In this study, we analysed the country-specific socioeconomic and health-related factors associated with disability among people aged 50 years and older (50+) in six LMICs.

## 2. Materials and Methods

Cross-sectional data from the WHO longitudinal multi-country Study on Global AGEing and Adult Health (SAGE) Wave 1, conducted in China, Ghana, India, Mexico, the Russian Federation, and South Africa during 2007–2010 was used for the analyses. These six countries were selected because they represent LMICs in four different world regions and are in different stages of economic development and of demographic and epidemiological transition [20]. In this study, the older population was defined as 50 years and older because in LMICs, the life expectancy at birth was 12 years lower than in the higher income countries. Thus, by applying the usual definition of older population (60 or 65+), the size of the older population would be much smaller [20].

WHO SAGE used multistage cluster sampling to generate nationally representative samples of people aged 50+ in each country. The response rate for respondents age 50+ for each country varied from 53% in Mexico to 93% in China [25]. SAGE collected information on sociodemographic characteristics, health behaviours, social networks, health status, and disability through face-to-face interviews. More detailed information on study design and methods have been published elsewhere [20,25].

### 2.1. Main Outcome Variable

The main outcome for this analysis is ADLs. Five questions from the World Health Organization Disability Assessment Schedule (WHODAS) II questionnaire were used for assessing any difficulties in performing daily activities in the preceding 30 days (such as bathing or washing the whole body, dressing, getting up from lying down, eating (including cutting up food), and getting to and using the toilet). The response was in a Likert scale format ranging from “without difficulty” to “with extreme difficulty”. In this study, respondents were considered as “with disability” if they reported any difficulties in performing at least one of the five daily activities listed above [26].

### 2.2. Covariates

#### 2.2.1. Sociodemographic Factors

Age was grouped in ten-year age categories (50–59, 60–69, 70–79, and 80+). Marital status was dichotomised into “partnered” (including those who were married or cohabiting) and “not partnered” (those who had never married and those who were divorced, separated, or widowed). Education level was grouped into high level (university), middle level (high school and secondary school), and low level (primary, less than primary level, and no formal schooling).

We define social capital as the quality, quantity, and degree of the connectedness of social relations [27]. Individual social capital was assessed based on access to two forms of social capital, structural and cognitive, in the preceding 12 months. Structural social capital refers to the extent and intensity of one’s social network and participation [27]. Questions on bonding, bridging, and linking structural social capital asked how often respondents participated in any social activities. Cognitive social capital includes perception of support, norms of trust, and reciprocity [27]. It was measured through questions on general trust, personal trust, and safety. More details on how the social capital variable was derived have been reported elsewhere [28]. Respondents were then categorised into those with access to both dimensions of social capital, structural social capital only, cognitive social capital only, and those with no access to either structural or cognitive social capital. Location of residence was classified into urban and rural areas. Urban areas included any area that had been legally proclaimed as urban (towns, cities, and metropolitan areas) and rural areas included commercial farms, small villages, and other areas that were further away from towns and cities [20]. Socioeconomic status was classified based on household characteristics such as housing characteristics, household’s ownership of durable assets and access to water, sanitation, and types of cooking fuel. We created wealth quintile using principal component analysis (PCA), the 1st quintile of wealth status represents the poorest and the 5th is the wealthiest households.

#### 2.2.2. Health Behaviours and Chronic Conditions

Physical activity level was measured using the Global Physical Activity Questionnaire (GPAQ) consisting of 16 questions that measure the frequency and duration of physical activity in three domains including work, transport-related, and recreational. Metabolic equivalences (METs) were calculated from the questions, and based on the METs value and duration of activities [29,30], respondents were grouped into three groups of high, medium, and low level of physical activity.

Body Mass Index (BMI) (kg/m^2^) was calculated based on anthropometric measurements of weight (in kilograms) and height (in metres), and then grouped into underweight (<18.5 kg/m^2^), normal (18.5–24.9 kg/m^2^), overweight (25–29.9 kg/m^2^), and obese (≥30.0 kg/m^2^) [31].

Presence of chronic conditions—Respondents were asked if they had been diagnosed with any chronic conditions including arthritis, stroke, angina, diabetes, chronic lung disease, and asthma, and if they had taken medication for their condition(s) in the last 12 months. In addition, arthritis, angina, and asthma were also assessed by using a set of validated symptom-based questions [20]. Hypertension was determined if respondents had been taking medication in the last 12 months or the average of measured systolic blood pressure was ≥140 mmHg and/or diastolic blood pressure was ≥90 mmHg. Based on the presence of the chronic conditions above, respondents were grouped into three groups: no chronic condition, with one chronic condition, and with two or more chronic conditions (multimorbidity).

Depression was assessed using 18 questions derived from the World Mental Health Survey version of the Composite International Diagnostic Interview covering the presence of 10 depression symptoms within the prior 12 months. The International Classification of Diseases tenth revision (ICD-10) criteria of depression was used to determine respondents with major depressive disorder (depression) [32]. According to the ICD-10, assessment of depression uses two criteria: 1) Reported at least four of the 10 symptoms present for most of the day (almost every day) or lasting for more than two weeks, and 2) at least two of the following symptoms are present: depressed mood, loss of interest, and fatigability. In addition, respondents who reported taking any medication or other treatment for depression during the last 12 months were also categorised as having depression.

### 2.3. Statistical Analysis

A descriptive analysis was employed to explore participants’ characteristics and the prevalence of ADL disability in each country. The association of sociodemographic characteristics, health behaviours, social capital, and ADL disability in each country was examined using multivariable logistic regressions. The individual effect of each covariate was tested in a univariable model. Covariates with significant effects or having been shown to be associated with ADL disability were included in the final model. An individual-level post-stratification weight was used to adjust for population age and sex distributions in each country. We tested for multicollinearity as well as for interaction terms between sex, age, and health-related covariates. We found no significant multicollinearity, but we found some significant interaction terms in China, Ghana, and India. A significant interaction of age group with depression and number of chronic conditions was found in China. The interaction between sex and age group was only significant in India. In Ghana, the interaction terms between sex and BMI, and age groups and physical activity were significant. We added the interaction terms in the final model for those three countries, but there are no major differences in the results and interpretations. A goodness-of-fit test was conducted to understand how well the models fit our data. All analyses were performed using Stata 15.1 (StataCorp, College Station, TX, USA).

### 2.4. Ethical Consideration

The WHO Ethical Review Committee reviewed and approved the SAGE survey (RPC149). SAGE was also reviewed and received ethical clearance from implementing partner institutions in the respective countries.

## 3. Results

A total of 36,428 individuals aged 50 years and over participated in the WHO SAGE wave 1. We excluded 11% of respondents who had missing data in any variables that were included in this study. A total of 32,567 individuals were included in the subsequent analysis (14,834 men and 17,733 women). The sociodemographic characteristics of respondents are presented in Table 1.

Overall, the majority of respondents were women (except in Ghana and India), belonged to the youngest age group (50–59 years), were married or cohabiting, had a low education level (except in the Russian Federation), and had access to both structural and cognitive social capital (except in China). The majority of SAGE respondents had a high level of physical activity, except those in South Africa. The prevalence of obesity varied from 3.5% in India to 49.8% in South Africa. The Russian Federation had the highest prevalence of multimorbidity (45%) (Table 1). The most common chronic conditions reported by SAGE respondents were hypertension (25.8%) and arthritis (24.3%) (Table A1).

**Figure 1:**
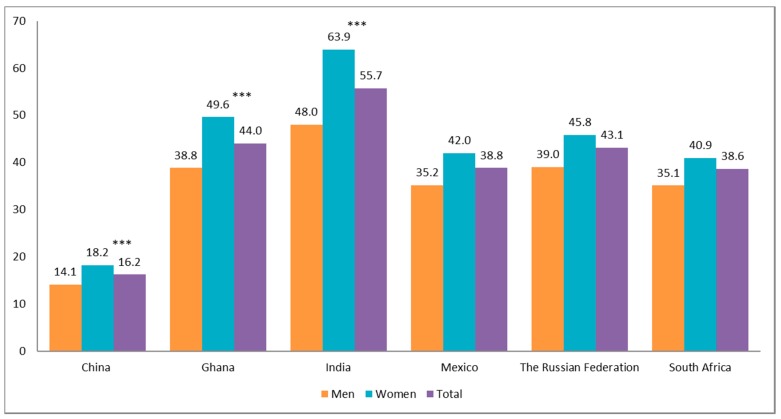
Prevalence of ADL disability among women and men aged 50+, by country, SAGE Wave 1 (2007–2010).

Note: The Chi-square test was used to assess the association between sex and ADL disability, (***) *p* < 0.001.

Figure 1 shows the prevalence of activities of daily living (ADL) disability among women and men aged 50+, by country. China had the lowest prevalence of disability (16.2%) in both men and women, in contrast to India (55.7%). Overall, women consistently reported a higher prevalence of disability compared to men in all countries.

Table 2 presents the associations between sociodemographic characteristics, health behaviours, social capital, and ADL disability by country, adjusting for other potential confounders. The mutual factors associated with ADL disability among six SAGE countries were age, presence of chronic conditions, and depression. The odds of reporting an ADL disability increased with age, particularly in the oldest age group (80+). Multimorbidity increased the odds of reporting an ADL disability by three to five times compared to participants without any chronic condition in all the six SAGE countries. Depression increased the odds of reporting an ADL disability by 1.5 times in Ghana to 2.5 times in China (*p* < 0.05).

Our findings reveal that factors associated with ADL disability varied across six SAGE countries. Women had higher odds of reporting an ADL disability than men in China (OR = 1.14, 95% CI: 1.01–1.29), Ghana (OR = 1.22, 95% CI: 1.01–1.48), and India (OR = 1.65, 95%CI: 1.37–1.99). Respondents with a low education level had higher odds of reporting a disability in almost all SAGE countries, except in Mexico and South Africa (*p* > 0.05). Having no partner was insignificantly associated with ADL disability, except in Mexico where older Mexicans who are not partnered were 50% less likely to report an ADL disability (95% CI: 0.30–0.83). Socioeconomic status was significantly associated with ADL disability only in China, India, and Mexico (*p* < 0.05), with respondents who resided in households with a low wealth quintile having increased odds of reporting an ADL disability. Older people who lived in a rural area in China and Mexico had higher odds of reporting an ADL disability (OR = 2.16, 95% CI = 1.73–2.69 and OR = 2.39, 95% CI = 1.16–4.92, respectively). Having no access to social capital increased the odds of reporting an ADL disability to about 2.6 times higher in China (95% CI: 1.54–4.31) and about 4 times higher in South Africa (95% CI: 1.79–9.43).

In addition, being overweight or obese was only significantly associated with ADL disability in Mexico (OR = 3.72, 95% CI = 1.93–7.17 for overweight and OR = 2.33, 95% CI = 1.27–4.27 for obesity) and in the Russian Federation (OR = 1.82, 95% CI = 1.09–3.04 for obesity). A low physical activity level increased the odds of ADL disability in all SAGE countries except in Ghana and South Africa (*p* > 0.05). For a moderate physical activity level, the odds were only significant in China and Ghana. In contrast, older people in South Africa with a moderate level of physical activity were 36% less likely to report an ADL disability (OR = 0.64, 95% CI = 0.43–0.96) compared to their counterparts with high level of physical activity.

## 4. Discussion

The main findings of this study show a diversity of determinants associated with ADL disability among older people aged 50 years and over in six SAGE countries. Our findings strengthen the evidence of the associations between sociodemographic characteristics, health behaviours, social capital, depression, and disability. In this study, significant gender effects were observed and varied across the six SAGE countries, however these effects were only significant in China, Ghana, and India. Previous studies have suggested that the higher prevalence of disability among women was due to a higher prevalence of chronic diseases [16] and especially non-fatal disabling health conditions [33]. Using the SAGE data, Williams et al. conducted a decomposition analysis of disability among older people aged 50+ in China and India. They found that gender inequality in disability in India was attributed predominantly to education levels, employment, and the presence of chronic disease [23]. It is also possible that there are some unexplained gender effects on ADL disability, such as gender-specific sociocultural factors that explain why women are more likely to develop an ADL disability in some countries. Further study is needed to explore the gender-specific factors.

Age and chronic conditions have been reported to be significant factors for ADL disability in previous studies [13,34,35]. In this study, the effect of age on disability could be related to physiological changes in body functions as people age. A study among older Brazilian people aged 60+ showed that functional decline was apparent even among respondents without any cognitive impairment and medical problems [36]. In addition, the higher prevalence of disability in older populations could be related to the higher prevalence of chronic conditions in this population. Consistent with findings from other studies in Spain [13], Brazil [34], and Canada, our findings show that among health-related factors, the number of chronic conditions has the strongest effect on ADL disability. Unlike physical activity or BMI, the presence of chronic diseases has a direct effect on people’s ability to perform ADL tasks, as chronic diseases can lead to a deterioration of body structure and function [37]. 

Regarding access to social capital, our findings echo previous studies in Japan and Brazil, which found that limited access to social capital was associated with ADL disability among the older population [18,38]. Using similar data (WHO SAGE data), Ng and Eriksson reported a positive association between access to social capital and self-reported health among older people in these SAGE countries [28]. The effects of access to social capital on ADL disability are more likely indirect. Structural social capital (bonding, bridging, and linking) has a significant role in preventing depression [39]. In general, access to social capital could increase access to health information which in turn helps in sustaining positive health behaviours [40,41,42].

The diverse association observed between household wealth and ADL disability across the six SAGE countries could be moderated by the different patterns of chronic disease risk factors, multimorbidity, and access to health care and health prevention program observed among older people in these countries. Irrespective of socioeconomic status, older people in lower income countries were more likely to have higher BMI, live a sedentary lifestyle, and smoke. In a previous study examined the socioeconomic inequalities of chronic disease risk factors in LMICs using data from the World Health Survey [43], the researchers showed that the patterns of chronic disease risk factors differed across sexes and income groups. As countries grew richer, adoption of healthy behaviour increased in all wealth strata [43]. This association could lie in the pathway between socioeconomic factors and disability observed in our study.

Physical inactivity was strongly associated with ADL disability among the older population in LMICs, which is in line with other previous studies [13,14,42]. The protective effect of physical activity on ADL disability results from complex pathways and is likely multifactorial [14]. For example, being physically active has been shown to reduce inflammation biomarkers, thus preventing the progression of chronic diseases. In addition, physical activity may increase social interactions, which could prevent depression. Both of these pathways may prevent disability [14].

Our studies also confirmed the association between depression and physical limitation shown in previous studies [44,45]. Some theories suggest that depression could result in disability directly and indirectly. Long-term depressive symptoms such as lack of sleep and appetite may directly cause functional decline. Depression may also lead to unhealthy behaviours (e.g., smoking, lack of exercise) that in turn cause disability [44]. On the other hand, disability may limit one’s activities and reduce their social interaction, possibly leading to depression [44,46]. 

Studies have shown that overweight and obesity were significantly associated with disability among older population [10,11], which is consistent with our findings. The association between BMI and ADL disability could be indirect as a high BMI is a known risk factor for chronic conditions, and chronic conditions increase the risk of disability [47,48]. Obesity is also a risk factor for falling among older people, which could result in physical limitations [49]. In addition, severe obesity could cause a general sense of fatigue and motor limitations that could negatively affect one’s ability to perform ADLs [50].

### Strengths and Limitations of the Study

Even though the associations between sociodemographic, health behaviours, and disability are well known in high-income countries, cross-country comparisons using harmonised instruments in LMICs are scarce. Our study is among the first study to fill this gap. The WHO SAGE employed a multistage cluster sampling design to generate a nationally representative sample. Furthermore, standardised instruments were used in all SAGE countries. These methodological measures aim to increase the external validity of findings in this study to the target population and ensure comparability of the findings across the SAGE countries [25]. In addition, our study tested various potential factors of ADL disability, including socioeconomic, social capital, physical and mental health, thus allowing for a more comprehensive outlook on factors associated with disability.

This study has several limitations. First, due to the cross-sectional nature of the data, it is not possible for us to ascertain the causal associations. Poor access to social capital can lead to disability among older people, but those with a disability are also at higher risk of having lower access to social capital—an example of reverse causation that could not be addressed in this study. Further investigation using panel data from the WHO SAGE when the 2nd Wave of the dataset is publicly available could address this problem. Second, the current study used BMI as a nutritional status measure in an older population. Even though BMI is the most commonly used measure, we acknowledge that there is controversy over whether BMI is an appropriate measure for older people as older people may experience changes in their stature and body composition (muscle loss) due to natural physiological changes or diseases [51,52,53]. Third, most of the variables in the WHO SAGE, including the disability questions, are self-reported. This may have led to overestimation or underestimation of the true prevalence of ADL disability among older people. Future studies should attempt to measure the prevalence of disability by combining the subjective measurements of ADL disability with more objective measurements of disability such as walking speed and the grip strength test. 

## 5. Conclusions

The factors associated with self-reported ADL disability among older people aged 50+ vary across the six SAGE countries. Age, presence of chronic conditions, and depression are common factors related to disability among the older population in all SAGE countries. Identifying determinants associated with ADL disability among older people in LMICs, which are currently at different stages of epidemiological transition and have different health systems, can help inform how health prevention programmes can best be implemented considering different country-specific factors.

## Figures and Tables

**Table 1:** Sociodemographic characteristics of respondents and distribution of body mass index, physical activity level, and self-reported chronic disease among respondents aged 50+, by country, WHO Study on Global AGEing and Adult Health (WHO SAGE) Wave 1.

Sociodemographic variables		China (n = 12,085)	Ghana (n = 4057)	India (n = 6257)	Mexico (n = 3940)	Russian Federation (n = 3422)	South Africa (n = 2806)
Gender (%)							
	Men	49.3	52.5	51.2	46.3	39.7	40.7
	Women	50.7	47.5	48.8	53.7	60.3	59.3
Age group (%)							
	50–59 years	45.2	40.5	49.2	50.7	46.8	49.3
	60–69 years	32.0	27.4	30.9	25.4	24.7	31.0
	70–79 years	18.5	22.8	15.7	17.0	21.6	14.3
	80+ years	4.2	9.3	4.2	6.9	6.9	5.3
Marital status (%)							
	Partnered	85.3	59.4	77.5	73.9	59.4	52.3
	Not currently partnered	14.7	40.6	22.5	26.1	40.6	47.7
Education level (%)							
	High	3.9	3.7	5.2	7.7	17.7	6.1
	Medium	31.9	21.6	19.2	12.6	75.2	22.5
	Low	64.1	74.7	75.6	79.7	7.1	71.4
Access to social capital (%)							
	Structural and cognitive	36.9	88.9	60.7	45.4	37.1	55.5
	Structural only	0.5	5.3	5.5	11.1	16.9	39.3
	Cognitive only	61.2	5.5	29.9	36.3	29.0	2.8
	Neither	1.3	0.4	3.9	7.2	17.0	2.4
Wealth status (%)							
	5^th^ quintile	20.6	21.3	24.0	27.1	24.5	21.5
	4^th^ quintile	23.6	21.0	19.7	16.0	21.8	18.6
	3^rd^ quintile	20.6	20.4	19.0	16.3	18.8	19.8
	2^nd^ quintile	18.7	19.0	19.0	25.6	19.5	20.4
	1^st^ quintile	16.5	18.3	18.3	15.0	15.5	19.7
Area of Residence (%)							
	Urban	46.0	40.7	25.7	78.0	72.5	65.3
	Rural	54.0	59.3	74.3	22.0	27.5	34.7
Body Mass Index (%)							
	Underweight	6.3	19.3	44.1	1.0	1.6	4.6
	Normal	58.5	51.2	43.0	20.7	23.1	23.1
	Overweight	26.5	16.6	9.3	40.8	33.7	22.4
	Obese	8.8	12.9	3.5	37.5	41.6	49.8
Physical activity level (%)							
	High	45.8	63.1	53.3	40.9	60.5	29.2
	Moderate	26.8	12.4	23.1	23.0	16.0	13.6
	Low	27.4	24.5	23.7	36.0	23.5	57.2
Chronic conditions (%)							
	None	51.8	54.1	44.9	44.7	27.9	46.9
	1 chronic condition	30.8	29.4	29.9	31.7	26.8	29.8
	Multimorbidity	17.4	16.6	25.2	23.6	45.3	23.4
Depression (%)		2.0	9.1	19.1	14.3	7.0	5.5

**Table 2:** Multivariable logistic regression of the association between sociodemographic factors, social capital, health behaviours, and ADL disability among respondents aged 50+, by country, SAGE Wave 1.

		Odds Ratio (95% CI)					
		China	Ghana	India	Mexico	Russian Federation	South Africa
Gender	Men	1	1	1	1	1	1
	Women	1.14 * (1.01–1.29)	1.22 * (1.01–1.48)	1.65 *** (1.37–1.99)	1.26 (0.75–2.10)	0.77 (0.46–1.27)	1.00 (0.76–1.31)
Age group	50–59	1	1	1	1	1	1
	60–69	1.49 *** (1.24–1.79)	1.40 *** (1.16–1.71)	1.31 ** (1.08–1.59)	1.05 (0.63–1.76)	1.73 * (1.04–2.86)	1.12 (0.84–1.49)
	70–79	2.60 *** (2.06–3.29)	2.45 *** (1.94–3.08)	1.87 *** (1.44–2.43)	1.43 (0.90–2.28)	3.50 *** (1.74–7.04)	2.10 *** (1.48–2.97)
	80+	4.99 *** (3.66–6.80)	3.89 *** (2.79–5.44)	3.24 *** (2.17–4.83)	3.52 *** (1.71–7.22)	8.52 *** (4.27–17.0)	3.63 *** (2.05–6.44)
Marital status	Partnered	1	1	1	1	1	1
	Not partnered	0.99 (0.83–1.17)	1.16 (0.95–1.41)	1.01 (0.82–1.24)	0.50 ** (0.30–0.83)	1.44 (0.97–2.14)	1.21 (0.89–1.63)
Education level	High	1	1	1	1	1	1
	Medium	1.39 (0.90–2.13)	1.20 (0.76–1.90)	1.79 ** (1.20–2.67)	0.94 (0.23–3.76)	1.80 * (1.15–2.80)	1.60 (0.76–3.39)
	Low	1.91 ** (1.30–2.79)	1.58 * (1.01–2.47)	2.54 *** (1.71–3.79)	1.28 (0.43–3.78)	3.54 *** (1.69–7.42)	1.79 (0.84–3.79)
Access to social capital	Both	1	1	1	1	1	1
	Structural only	0.78 (0.25–2.45)	0.86 (0.46–1.62)	0.99 (0.67–1.46)	0.69 (0.30–1.56)	2.21 * (1.08–4.55)	1.36 * (1.03–1.81)
	Cognitive only	1.07 (0.83–1.38)	0.83 (0.51–1.35)	0.80 * (0.65–1.00)	2.54 *** (1.54–4.21)	1.59 * (1.09–2.31)	1.84 (0.91–3.73)
	Neither	2.57 *** (1.54–4.31)	1.24 (0.39–3.97)	1.21 (0.81–1.79)	1.03 (0.36–2.92)	1.68 (0.88–3.24)	4.11 *** (1.79–9.43)
Wealth status	1^st^ quintile	2.03 *** (1.39–2.96)	1.23 (0.88–1.70)	1.59 ** (1.20–2.11)	1.96 * (1.01–3.83)	1.11 (0.73–1.69)	0.95 (0.57–1.59)
	2^nd^ quintile	1.91 ** (1.31–2.77)	1.34 (0.99–1.82)	1.45 ** (1.15–1.83)	0.80 (0.37–1.72)	1.13 (0.71–1.81)	0.93 (0.60–1.45)
	3^rd^ quintile	2.24 *** (1.63–3.08)	1.86 *** (1.40–2.47)	1.50 ** (1.13–1.98)	1.74 (0.84–3.60)	0.98 (0.58–1.67)	0.75 (0.49–1.16)
	4^th^ quintile	1.65 ** (1.19–2.29)	1.09 (0.85–1.39)	1.28 (0.99–1.65)	0.73 (0.37–1.43)	0.49 ** (0.29–0.82)	0.76 (0.54–1.07)
	5^th^ quintile	1	1	1	1	1	1
Residence	Urban	1	1	1	1	1	1
	Rural	2.16 *** (1.73–2.69)	1.12 (0.83–1.50)	0.94 (0.75–1.19)	2.39 * (1.16–4.92)	0.98 (0.63–1.51)	1.13 (0.81–1.58)
Body mass index (BMI)	Underweight	0.90 (0.74–1.08)	1.09 (0.86–1.39)	1.16 (0.93–1.43)	1.64 (0.43–6.27)	1.96 (0.75–5.13)	0.93 (0.52–1.67)
	Normal	1	1	1	1	1	1
	Overweight	0.86 (0.74–1.00)	0.97 (0.77–1.21)	1.12 (0.87–1.45)	3.72 *** (1.93–7.17)	0.95 (0.56–1.59)	0.81 (0.57–1.15)
	Obese	1.00 (0.81–1.23)	1.06 (0.83–1.34)	1.12 (0.68–1.85)	2.33 ** (1.27–4.27)	1.82 * (1.09–3.04)	0.95 (0.69–1.31)
Physical activity	High	1	1	1	1	1	1
	Moderate	1.38 *** (1.17–1.63)	1.37 * (1.02–1.82)	1.20 (0.99–1.45)	1.82 (0.91–3.64)	1.09 (0.75–1.59)	0.64 * (0.43–0.96)
	Low	2.13 *** (1.74–2.61)	1.17 (0.87–1.59)	1.70 *** (1.38–2.10)	1.94 * (1.11–3.38)	2.20 ** (1.64–2.96)	1.03 (0.75–1.43)
Mental health	No Depression	1	1	1	1	1	1
	Depression	2.54 *** (1.82–3.54)	1.51 * (1.05–2.15)	2.49 *** (1.99–3.11)	2.28 * (1.20–4.33)	1.05 (0.58–1.87)	1.90 ** (1.20–3.00)
Presence of chronic disease	None	1	1	1	1	1	1
	One chronic disease	1.59 *** (1.28–1.96)	1.57 *** (1.30–1.89)	1.72 *** (1.46–2.03)	1.72 (0.98–3.02)	1.82 * (1.13–2.93)	1.69 *** (1.25–2.30)
	Multimorbidity	2.91 *** (2.43–3.47)	3.18 *** (2.42–4.18)	2.64 *** (2.08–3.36)	3.44 *** (1.92–6.17)	4.72 *** (2.54–8.79)	2.83 *** (1.91–4.18)

Note: the significant values of *p* < 0.05 (*), *p* < 0.01 (**), *p* < 0.001 (***).

## Appendix A

**Table A1:** Prevalence of chronic conditions among respondents aged 50+, by country, WHO SAGE Wave 1.

Variables	China	Ghana	India	Mexico	Russian Federation	South Africa	All countries
Angina (%)	8.0	12.2	17	14	36.4	7.9	16.7
Stroke (%)	1.9	1.5	1.0	1.9	3.1	2.7	1.8
Asthma (%)	3.9	3.7	10.8	3.7	5.9	6.6	7.0
Chronic lung disease (%)	4.9	0.3	2.2	0.6	6.8	1.6	4.1
Diabetes (%)	6.5	3.8	6.8	17.5	6.9	9.4	6.7
Hypertension (%)	25.2	13.1	14.1	25.4	51.3	30.0	25.8
Arthritis (%)	20.5	23.5	23.7	12.3	33.5	26.3	24.3
Depression (%)	2.0	9.1	19.1	14.3	7.0	5.5	9.6
